# Veterinary pharmacovigilance in sub-Sahara Africa context: a pilot study of adverse reactions to veterinary medicine in Cameroon

**DOI:** 10.1186/s12917-019-2043-1

**Published:** 2019-08-19

**Authors:** Mohamed Moctar Mouliom Mouiche, Badou Zaki Ndouoya Njingou, Frédéric Moffo, Serge Eugene Mpouam, Jean Marc Kameni Feussom, Julius Awah-Ndukum

**Affiliations:** 1 Department of Pharmacy, Pharmacology and Toxicology, School of Veterinary Medicine and Sciences, University of Ngaoundere, Ngaoundere, Cameroon; 2 MOSAIC, Yaoundé, Cameroon; 3 Cameroon Epidemiological Network for Animal Diseases (RESCAM), Directorate of Veterinary Services, Ministry of Livestock, Fisheries and Animal Industries (MINEPIA), Yaoundé, Cameroon; 4 College of Technology, University of Bamenda, Bamenda, Cameroon

**Keywords:** Pharmacovigilance, Veterinary medicine, Adverse drugs reactions, Inefficacy, Reporting, Cameroon

## Abstract

**Background:**

Sub-Saharan African market is highly affected by counterfeit veterinary drugs. Though these counterfeit and non-compliance of drugs can induce adverse effects during their utilization, there is no monitoring system of veterinary medicines. The present pilot study was carried out in Cameroon to identify and describe suspected cases of adverse reactions to veterinary drugs in animals and / or humans as well as inefficacy of veterinary drugs. The methodology involved a descriptive cross-sectional survey of 67 actors in the veterinary medicine sector in Cameroon.

**Results:**

A total of 74/120 (62%) cases of suspected adverse effects and or lack of efficacy of veterinary drugs in animals and 46 (38%) cases of adverse reactions in humans were identified. Antiparasitics were the most incriminated therapeutic class in animals (61%) and human (56%). Adverse reactions were reported in dogs (44%) and poultry (24%) while drug inefficacy was most observed in poultry (47%). According to animal health professionals, levamisole (24%) and ivermectin (16%) were identified to be responsible for the adverse effects and that the highest level of inefficacy was most frequently reported for oxytetracycline (29%). The main adverse reactions were systemic (22%), gastrointestinal (20%) and neurological (13%) disorders.

**Conclusion:**

The results of this study showed that misuse and circulation of poor quality as well as lack of efficacy of veterinary drugs is very common in Cameroon. Adverse reactions were observed in animals and humans. Therefore, the establishment of a national veterinary pharmacovigilance system based on solid legal bases is essential for a continuous assessment of the risks-benefits effects of veterinary drugs marketed in Cameroon.

**Electronic supplementary material:**

The online version of this article (10.1186/s12917-019-2043-1) contains supplementary material, which is available to authorized users.

## Background

Animal diseases represent a permanent danger for pets and food producing animals and constitute a limit for livestock development which is of socio-economic importance in Sub-Saharan African countries including Cameroon. Indeed, at least 90% of livestock diseases on the list of the World Organization for Animal Health (OIE) are present in Africa [1] necessitating widespread use of veterinary drugs. However, chemoprevention and chemotherapy used to control animal diseases are not without risks for the treated animals [2] and can cause iatrogenic injury to the user [3, 4]. A lower efficacy of veterinary medicinal products than that provided in the Summary of Product Characteristics may be observed [4]. The misuse and non-respect of withdrawal periods can cause the presence of active residues in foodstuffs of animal origin [5] and ecological risk [6].

The marketing authorization folder, equivalent to the birth certificate of a drug, guarantees only its quality, safety and efficacy, since different studies for approval are done on a limited number of individuals and under controlled conditions [7, 8]. Surveillance systems are therefore very essential given that the profile of side effects and problems associated with the use of drugs may differ from one country to another, and even between regions within the same country.

Previous studies in sub-Saharan Africa on the concept of veterinary pharmacovigilance revealed that there is no real monitoring system [9] and legal vacuums prevail in many countries including Cameroon. For example, a cross-sectional descriptive survey in Senegal showed that 58% of veterinary practitioners have no idea about the concept of pharmacovigilance [10]. Joubert and Naidoo [11] in South Africa also reported low (42%) knowledge scores of pharmacists as concerns the concept of Pharmacovigilance. An assessment of pharmacovigilance systems in 26 sub-Saharan African countries showed that only 8 (30%) countries collected reports on adverse events with only 3 programs to contribute a sizeable number of reports. Of the eight countries collecting adverse events, seven were members of the WHO Programs for International Drug Monitoring (PIDM), and in those countries where a PV system existed, it was not well integrated with other regulatory activities [12]. In Cameroon, as concerns regulation of pharmacovigilance, a sub commission of PhV and vaccinovigilance was created by the decree N°2008/2909 on December 08, 2008 and it still not functioning up today [13]. Due to the irrational use of drugs, the circulation of counterfeit medicines and the poor drug regulation in the pharmaceutical sector [14–17] , the establishment of important surveillance systems for post-marketing monitoring of drugs in veterinary practice cannot be overemphasized. In the context of promoting veterinary pharmacovigilance, this study was carried out to identify cases of suspected adverse reactions (ADRs) of veterinary drugs in animals and / or humans and cases of inefficacy of veterinary drugs in Cameroon.

## Results

### Adverse drug reactions and lack of efficacy observed in animals

In the cross-sectional survey, 120 cases of adverse reactions and / or lack of efficacy that occurred from January 2001 to May 2014 were notified (Table 1). These cases are distributed as follows: 46 (38%) cases of adverse effects in animals, 28 (24%) cases of inefficacy in animals and 46 (38%) cases of adverse effects in humans.

**Table 1:** Distribution of cases of adverse event due to veterinary medicinal products

	Cases of adverse reactions and / or lack of efficacy			
	ADR in animal	ADR in human	Lack of efficacy	
Years				Overall
2001–2005	8	4	2	14
2006–2010	8	11	5	24
2011–2014	30	31	21	82
Total	46	46	28	120

**Fig. 1:**
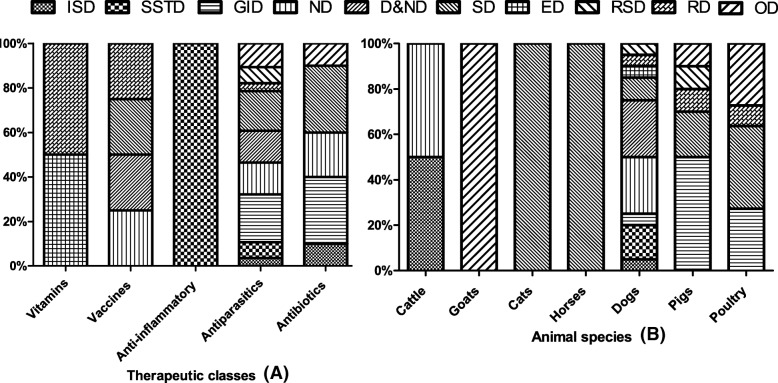
Distribution of ADRs observed in animal with respect to therapeutics classes (**a**) and animal species (**b**). *ISD*: Immune system disorders; *SSTD*: Skin and subcutaneous tissue disorders; *GID*: Gastrointestinal disorders; *ND*: Neurological disorders; *SD*: Systemic disorders; *ED*: Eye disorders; *RD*: Respiratory disorders; *RSD*: Reproductive system disorders; *DND*: Digestive and neurological disorders; *OD*: Others disorders

The main species that manifested adverse effects were dogs (44%), poultry (24%) and pigs (22%). The lack of efficacy of veterinary drugs was encountered mainly in poultry (47%) and cattle (21%) (Table 2). Antiparasitics (61%) were the most reported class of drugs associated with cases of adverse reactions in animals, followed by antibiotics (24%) (Fig. 1a). The inefficacy of veterinary drugs was mostly encountered when using antibiotics (68%) (Table 3). The incriminated molecules for adverse reactions were mainly levamisol (24%), ivermectin (16%), vaccines (11%) and oxytetracycline (9%). In the case of inefficacy, oxytetracycline (29%) was the most frequently reported (Table 4). According to the terms of use, adverse effects occurred in 52% of cases following the use of veterinary medicinal products in accordance with the manufacturer’s instructions. In 35% of cases, adverse effects occurred after medication was used in non-compliance with the manufacturer’s instructions and no information was available in 13% of cases on how to use veterinary drugs. Also, drug inefficacy was suspected in 82% of the cases after administering medication according to the manufacturer’s instructions and off-label in 11% of the cases. No information was available in 7% of the cases on how to use veterinary drugs.

**Table 2:** Distribution of ADRs and lack of efficacy with respect to species

Animal species	Presumed adverse drug reaction (%) (*n* = 46)	Lack of efficacy (%) (*n* = 28)
Cattle	4	21
Goats	2	/
Cats	2	/
Dogs	44	18
Horses	2	/
Pigs	22	14
Poultry	24	44

**Table 3:** Distribution of ADRs and lack of efficacy with respect to therapeutic classes

Therapeutic classes	Presumed adverse drug reaction (%) (*n* = 46)	Lack of efficacy (%) (*n* = 28)
Antibiotics	24	68
Antiparasitics	61	14
Anti-inflammatory drugs	2	/
Vaccines	11	7
Anticoccidials	/	7
Anaesthetics	/	4
Others	2	/

**Table 4:** Distribution of ADRs and Lack of efficacy with respect to the molecule

Molecules	ATCvet Code	Presumed adverse drug reaction (%) (*n* = 46)	Lack of efficacy (%) (*n* = 28)
Levamisol	QP52AE01	24	/
Oxytetracycline	QJ51AA06	9	29
Cypermethrin	QP53AC08	9	7
Vaccines	QI	11	7
Vitamines	QA11BA	2	/
Diminazen	QP51AF01	2	/
Furaltadone	QJ01XX93	4	/
Flumequin	QJ01MB07	2	4
Deltamethrin	QP53AC11	7	/
Praziquantel	QP52AA51	2	/
Sulfadimidine	QJ01EQ03	2	7
Ivermectin	QD11AX22	16	7
Cortisone	QH02AB10	2	/
Dimpylate	QP53AF03	2	/
Colistine	QA07AA10	2	/
Doxycycline	QJ51AA02	2	/
Benzylpenicillin- streptomycin	QJ51CE59	/	7
Norfloxacin	QJ01MA06	/	7
Acepromazin	QN05AA04	/	4
Streptomycin	QJ01GA01	/	4
Benzylpenicillin	QJ51CE01	/	4
Amoxicillin	QJ01CA04	/	4

### Types of adverse events observed and their evolution

Table 5 shows that, the observed adverse reactions were mainly systemic disorders (22%), gastrointestinal disorders (20%) and neurological disorders (13%). These side effects resulted in death (52%), and 31% of adverse reactions cases healed without apparent follow-up while 17% healed with follow-up. Dogs presented all the types of adverse effects, followed by pigs and poultry who presented 5 and 4 types respectively (Fig. 1b).

**Table 5:** Types of ADRs observed in animals

SOC of ADRs observed	*n* = 46
Immune system disorders	4%
Skin and subcutaneous tissue disorders	6%
Gastrointestinal disorders	20%
Neurological disorders	13%
Systemic disorders	22%
Eye disorders	2%
Respiratory disorders	7%
Reproductive system disorders	4%
Digestifs and neurological disorders	11%
Others (NS)	11%

*SOC* system organ class, *NS* not specified

**Fig. 2:**
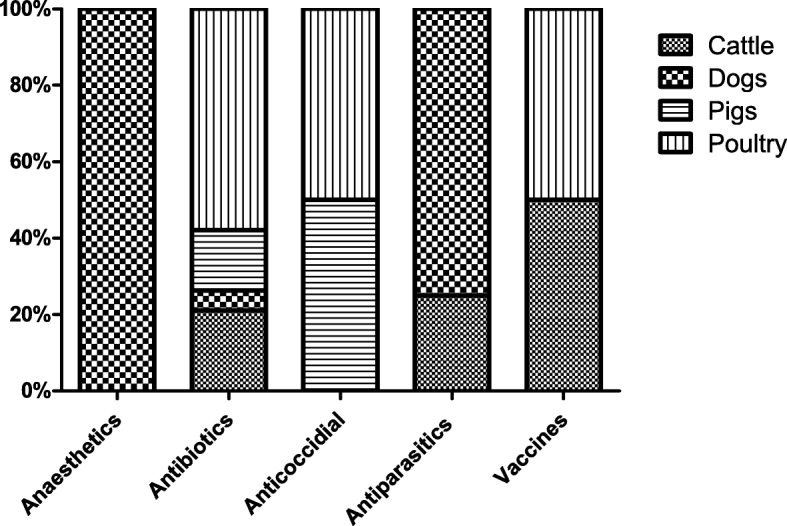
Distribution of lack of efficacy with respect to animal species and therapeutic classes

Ineffective treatment resulted mostly from inadequate therapeutic effect at usual doses (39%) and the lack of therapeutic effect (29%). This ineffectiveness of drugs in 61% of cases resolved without follow-up after treatment. The information was not available for 18% of the cases while 14% of the cases were cured with follow-up with change of treatment. The ineffectiveness of antibiotics was observed in virtually all species. Antiparasitics were suspected of being ineffective in cattle and dogs and vaccine inefficacy was observed mainly in poultry and dogs (Fig. 2).

### Adverse reactions observed in humans

A total of 46 cases of adverse reactions were reported in humans, specially by animal health professionals and or animal owners following the use of veterinary drugs. Antiparasitics (56%) and vaccines (31%) were the most incriminated therapeutic class for adverse reactions. The reported adverse reactions were respiratory disorders (19%), skin and subcutaneous disorders (19%), and others (31%) including fever, headache, stiffness and severe pain. The routes of exposure were mostly cutaneous (37%), subcutaneous (31%), respiratory (19%) and ocular (13%). The evolution of adverse reactions towards healing was without follow-up in 80% of the cases and with follow-up in 20% of cases.

### Notification of adverse reactions of veterinary drugs

About 55% of adverse reactions were reported in animals or humans and nearly 54% of the presumed inefficacy was reported by respondents. These cases of side effects or presumed ineffectiveness were reported either to the veterinary clinicians, the wholesaler-importers, the local representatives of the pharmaceutical company and to a lesser extent to the National Veterinary Council. The reasons given by stakeholders to justify under-investigation and underreporting of adverse events and/or inefficacy of veterinary drugs observed in animals were: (i) Ignorance on what to do (20%); (ii) Doubt on the role of the drug (40%) and (iii) the absence of national regulations on the reporting of ADRs and veterinary pharmacovigilance systems (40%). In total, 98.5% of respondents stated that it was important to report suspected adverse reactions and / or suspicions of inefficacy of veterinary drugs. The reasons given by these respondents were mainly corrective measures by competent authorities for the improvement of veterinary medicines in the market as well as the protection of animal health and public health.

## Discussion

Overall, 120 cases of adverse reactions and / or inefficacy of veterinary drugs were recorded over a period of 13 years from 2001 to May 2014. This finding does not seem to reflect reality and could be higher. Indeed, some respondents interviewed were negligent and remembered only recent cases. Indeed, they did not remember cases of adverse effects and / or alleged inefficacy of veterinary drugs and were not sure of the existence of a causal link between veterinary drugs and the occurrence of adverse effects. In pharmacovigilance, it is important that health professionals report all adverse effects, even if they are not sure that the veterinary drug in question is the real cause [7, 17]. Yet other stakeholders were reluctant to disclose cases of adverse effects and / or drug inefficacy for fear of how their employers would react. Under-reporting of ADRs cases is a crucial problem in pharmacovigilance over the world, but the situation is more observed in Africa. Ampadu et al. [18] indicate that at the end of September 2015, individual case safety reports (ICSRs) to VigiBase represented 0.88% of global ICSRs. Under-reporting of ADRs cases, may also due to the lack of pharmacovigilance center, lack of easier reporting forms or unavailability of reporting forms as previously reported by De Briyne et al. [19] in Europe and Syed et al. [20] in Pakistan.

However, a national veterinary pharmacovigilance system in Cameroon to monitor drugs can be set up based on the cases reported and their side effects to ensure accountability including traceability of all imported drugs in the country. Over 24% of the 120 cases of adverse reactions of veterinary drugs reported were due to suspected inefficacy while 38% were cases of adverse reactions in animals and 38% in man. Similar studies recorded 68% suspected drug inefficacy and 32% ADRs of 420 cases between 1997 to 2010; 16% drug inefficacy suspicions and 79% of ADRs of 376 cases from 1985 to 2011 and 43.30% of ADRs and 56.70% of suspicions of ineffective veterinary drugs in 443 cases between 1990 to 2011 respectively in Senegal [10, 21], Ivory Coast [22] and Burkina Faso [23]. This high proportion of ineffective drugs recorded in this study may be due to the invasion of many African countries including Cameroon with huge amounts of counterfeit drugs and mediocre generics [9, 11, 17]. Studies in Cameroon on the quality of veterinary drugs in the formal and non-formal sector gave a non-compliance rate of 69% [24] and 72% [25] which confirmed the hypothesis of Van Gool about the poor quality of veterinary drugs in Sub Saharan African market [9]. However, in France and other developed countries, the movement of poor quality veterinary drugs is hampered by strict procedures for marketing authorisation and market surveillance. Hence the importance of establishing a national veterinary pharmacovigilance system for monitoring of veterinary drugs after marketing authorization through monitoring of cases of ADRs and conducting accountability investigations.

The adverse reactions reported in this study occurred mainly in dogs (44%) and poultry (24%) since clinical activities in towns were oriented mostly to pets and an increase in poultry production in Cameroon. This distribution of adverse events by animal species is similar to that obtained by Naidoo and Sykes [26] in South Africa, Coulibaly [22] in Ivory Coast, Assoumy et al. [10] in Senegal and Davis et al. [27] in Britain who showed that dogs were the main species affected by ADRs after administration of veterinary drugs at proportions of 60, 51, 39 and 41%, respectively. Also, intense clinical activities are oriented towards canine medicine in these countries resulting in a high incidence of adverse drug effects in these species.

The main therapeutic classes suspected of causing adverse effects were antiparasitics and antibiotics. Antiparasitics and antibiotics are the therapeutic classes most sold in the Cameroonian market [25] and the most used in the treatment of domestic animals. These therapeutic classes of veterinary drugs are often subjects of counterfeiting and high rates of non-compliance in the Cameroonian market. The presence of these non-compliances and use of veterinary drugs accounted for the high adverse reactions reported. Also, the misuse of drugs or non-compliance of doses (overdose and under dose) can cause the occurrence of adverse events. Levamisole has a narrow therapeutic window and was the most incriminated molecule in this study. Indeed, Fatima [23] in Burkina Faso and Assoumy et al. [10] in Senegal had observed similar results where, antiparasitics and antibiotics were the most incriminated therapeutic classes. Suspected adverse reaction due to veterinary drugs resulted in death of 52% cases of affected animals with consequent enormous loss to pet owners.

The drug inefficacy identified in this study was in poultry [47%] and cattle (21%) due to improper use of veterinary drugs by animal health professionals, non-compliance to recommended doses and increasing antibiotic resistance common in poultry farms. Indeed, Coulibaly [22] in Ivory Coast observed similar results, unlike Fatima [23] in Burkina Faso who stated that dogs (46%) were the main species that presented ineffectiveness of veterinary drugs. This difference in results may be justified by the fact that the poultry industry was not well developed in Burkina Faso compared to ruminant farming.

The therapeutic classes associated with inefficacy are antibiotics (68%) and antiparasitics (14%). The finding agrees with Teko-agbo et al. [24] and Ndottiwa [25], who observed high rates of pharmaceutical noncompliance of veterinary drugs in Cameroon. Moreover, these classes are widely used in poultry farms especially antibiotics and their misuse could cause microbial resistance. In agreement with the report of Guetiya Wadoum et al. [28] oxytetracycline was the molecule most frequently reported and corresponded to the most widely used antibiotic in poultry farming. The abusive uses of antibiotics in farm exacerbate the phenomenon of antimicrobial resistance and lack of efficacy of veterinary drugs. Previous studies have reported high level of resistance to tetracycline in Cameroon. *Salmonella sp* isolated from chicken in the market showed 84.5% resistance to tetracycline [29] while lowest resistance rate of bacteria isolates from farm wastes to tetracycline of > 50% [30] have been reported. Guetiwa [28] also indicated that out of eleven different bacteria isolated from poultry farm in Cameroon, they were shows overall 63.6% resistance to oxytetracycline. Also, Akomoneh et al. [31] notified a rate of 71.4% resistance of ***Escherichia coli*** isolated from cattle to tetracycline in Cameroon.

Also, Coulibaly [22] in Ivory Coast and Assoumy et al. [21] in Senegal had identified antibiotics and antiparasitics as the origin of suspected inefficacy of veterinary drugs and that the use of these drugs according to instructions was respected in over 82% of cases. However, ineffective treatment administered to animals encouraged the emergence of microbial resistance strains that can be transmitted from animals to humans by direct or indirect contact, limiting effective treatment of humans [10].

Non respect of posology (11%), poor clinical diagnosis and pre-existing parasitic or bacterial resistance could explain the suspected inefficacy of veterinary drugs. However, exclusion of the role of the drug, following careful analysis, had been done in France showing that drug’s effectiveness is not questioned even when 17% of declarations were related to suspected lack of efficacy [8, 21]. The establishment of a National Committee for veterinary pharmacovigilance in Cameroon to conduct investigations cannot be overemphasized.

The consequences of the ineffective treatment and adverse effects associated with the use of veterinary drugs do not only contribute to a significant loss due to the observed morbidity and mortality, but also to lower production due to the persistence or aggravation of post-healing effects. Financial loss is accentuated by the change of treatment and costs related to the purchase of drugs or payment for veterinary fees.

Adverse reactions observed in humans were mostly due to antiparasitics (56%). Antiparasitics was the most sold class and most commonly used in clinics for treatment, and they also recorded high rates of poor quality. Ndotiwa in [25] reported that 80% of ivermectin and 71% of albendazole selected in the market in Cameroon were non-compliance. Also, Têko-agbo et al. [24] reported that 100% of trypanocides and 52% of anthelmintics and endectocides over the country were non-compliance. The non-compliance was relative to low quantity of active ingredient. The use of sub therapeutics quantities of drugs creates favorable condition to resistance or could be led to the lack of efficacy [32–34]. The most common clinical use of antiparasitics was geared towards pest control which entails more manipulation and therefore higher exposure of humans. The typology of these side effects could be explained by the fact that the routes of exposure to the product are mostly dermal and inhalation. Allergies and respiratory problems were reported with disease progressed in 80% of the cases to healing without follow up. This high rate of recovery could be explained by the fact that the exposure time was not very high. However, in 14% of the cases, the disease evolved to healing with follow up. The distribution of these evolutions is different from that obtained by Coulibaly [22] in Ivory Coast who reported cases of adverse reactions in humans with progression to death after the treatment of adverse effects observed in 10.53% of cases.

Approximately half of cases of side effects recorded and nearly 54% of cases of suspected inefficacy of veterinary drugs in animals were reported to the Veterinarians, the wholesaler-importers or local representatives of the pharmaceutical companies. This low rate of notification of adverse drug reactions was due to a lack of contact with the veterinary authorities by actors of the veterinary drug industry. This lack of statistics of adverse reaction reports by veterinary authorities could be explained by the lack of adverse drug reaction reporting forms and ignorance of what to do or unwillingness, and especially the lack of a pharmacovigilance system for reporting of adverse drug reaction. Coulibaly [22] in Ivory Coast and Assoumy et al. [35] in Niger have shown that the low level of notifications made to the veterinary authorities, is due to lack of pharmacovigilance system. This prevails in many African countries including Cameroon. Reporting of ADRs is essential for the success of any pharmacovigilance system.

A few stakeholders in the veterinary medicine sector surveyed have knowledge on the notion of veterinary pharmacovigilance and it is therefore a new concept for the majority of these respondents. Similar findings were reported by Joubert et al. [11] in South Africa or Syed et al. [20] in Pakistan where pharmacists and physicians have shown few knowledge on the concept of pharmacovigilance. These actors need information and training about pharmacovigilance. It appears that the terminology ‘pharmacovigilance’ was not understood by the respondents. All stakeholders agree on the importance of reporting adverse reactions because it would enable the protection of animal health, public health, environment and the fight against drug resistance and drug residues in foodstuff of animal origin. These reasons are in line with the goals of veterinary pharmacovigilance which are to ensure the safety of veterinary medicines in animals, safety of people in contact with the veterinary medicinal products, safety of foodstuff of animal origin (from treated animals), the protection of the environment, and the surveillance of drug resistance [14].

## Conclusion

Veterinary pharmacovigilance allows permanent update of knowledge on drug safety, it has as objective to prevent and reduce risks associated with veterinary drugs [36]. Given the results of this study, the establishment of a veterinary pharmacovigilance system and legislation becomes imperative in Cameroon and sub-Saharan Africa in general. This system will enable continuous evaluation of the benefits / risks of veterinary drugs marketed in Cameroon and safeguards public health risk of adverse drug effects.

## Methods

### Study design and sampling

The data were collected from December 2013 to November 2014 in 5 main cities (Bafoussam, Bamenda, Douala, Ngaoundere and Yaounde) of Cameroon in which over 80% of stakeholders in the veterinary medicine sector are found.

The methodological approach consisted of a cross-sectional survey. The target population was made up of all animal health professionals in the study area. Briefly, a list of veterinarians installed in the public and private sector was obtained from the sub-department of pharmacy and private sector of the Ministry of livestock fisheries and animal industries in Cameroon and the survey completed in the field. Overall, 67 stakeholders (39 wholesalers-importers-distributors of veterinary medicines, 23 veterinarians in public and private sectors and 05 representative’s agents of veterinary pharmaceutical companies) were investigated. The questionnaire (Additional file 1) was developed according to a previous study done by Assoumy et al. [10]. The questionnaire written in French and English, was used to collect data on cases of side effects or alleged ineffectiveness of veterinary drugs in animals and / or humans. The information collected on animals concerned the date of occurrence, the affected animal (animal species, number of animals), the drug incriminated (trade name, manufacturer laboratory, the active ingredient, therapeutic class, use according to the respect of authorization or not), its route and duration of exposure, the description of the adverse reaction and its evolution. In humans, the data collected were the date of occurrence, gender, age and occupation of the person exposed, the drug incriminated (trade name, manufacturing laboratory, active ingredient, therapeutic class), its route and duration of exposure, the description of associated adverse reaction and its evolution.

### Data analysis

The data obtained (different cases of suspected adverse reactions and lack of efficacy of veterinary drugs that occurred in animals and in humans) were entered in Microsoft Excel spreadsheet 2010 (Microsoft Corporation, Redmond, WA, USA). A descriptive data analysis was performed to express the results as calculated frequencies.

## Additional file

Additional file 1: Questionnaire addressed to animal health professionals to record the cases of adverse effects due to veterinary drug in an animal or human**. (PDF 125 kb)**

## Data Availability

The datasets used and/or analyzed during the current study are available from the corresponding author on reasonable request.

## Abbreviations

ADRs: Adverse Drugs Reactions
ATCvet: Anatomical Therapeutic Chemical classification system for veterinary drugs
DND: Digestive and Neurological Disorders
ED: Eye Disorders
GID: Gastrointestinal Disorders
ISD: Immune System Disorders
ND: Neurological Disorders
NS: Not Specified
OD: Others Disorders
OIE: World Organization for Animal Health
RD: Respiratory Disorders
RSD: Reproductive System Disorders
SD: Systemic Disorders
SOC: System Organ Class
SSTD: Skin and Subcutaneous Tissue Disorders
USA: United States of America
