# Supplementary material for: Veterinary pharmacovigilance in sub-Sahara Africa context: a pilot study of adverse reactions to veterinary medicine in Cameroon
Source: BMC Vet Res. 2019 Aug 19;15:301. doi: 10.1186/s12917-019-2043-1 (PMC6701137; doi:10.1186/s12917-019-2043-1)
Supplement: Supplementary file 1 — Questionnaire addressed to animal health professionals to record the cases of adverse effects due to veterinary drug in an animal or human. (PDF 125 kb) [file 12917_2019_2043_MOESM1_ESM.pdf]

Additional file1 : Questionnaire addressed to animal health professionals to record the cases of adverse effects due to veterinary drug in an animal or human

Questionnaire   N°..……..

Date :…../……/……….

Identification

1. You are a?
   - ☐ Private veterinary doctor
   - ☐ Public veterinary doctor
   - ☐ Veterinary nurse
   - ☐ Other (specify) …………………………………………………………………………

1. Have you ever observed adverse effects of a veterinary drug in an animal after administration?
   - ☐ YES
   - ☐ NO

   If yes, kindly fill **form Nº1**

   Did you report them?
   - ☐ YES
   - ☐ NO

   If yes, to whom?
   - ☐ The ‘Directorate of Veterinary Service (DSV)
   - ☐ Wholesalers-Importers
   - ☐ National Veterinary Council
   - ☐ Local representatives of the Pharmaceutical industry
   - ☐ A depositary
   - ☐ Colleagues
   - ☐ Others (precise)… ………………………………………………………………………………

   If no, why?
   - ☐ Not sure that the adverse effects were due to the drug
   - ☐ There are no regulations on the notification of adverse effects
   - ☐ Unawareness on the attitude to take
   - ☐ Lack of feedback information
   - ☐ Other (precise)………………………………………………………………………

3- Have you noticed a lack of efficacy of a particular drug upon administration?
   - ☐ YES
   - ☐ NO

   If yes, kindly fill **form Nº2**

   Did you report the concerned lack of efficacy?

- [ ] YES
- [ ] NO

If yes, to whom ?

- [ ] The ‘Directorate of Veterinary Service (DSV)
- [ ] Wholesalers-Importers
- [ ] National Veterinary Council
- [ ] Local representatives of the Pharmaceutical industry
- [ ] A depositary
- [ ] Colleagues
- [ ] Others (precise)… ………………………………………………………………………………

If no, why ?

- [ ] Not sure it was due to the drug
- [ ] There are no regulations on the reporting of adverse effects
- [ ] Unawareness on the attitude to take
- [ ] Lack of information feedback

Other (precise)………………………………………………………………………………………

4- Have you ever had adverse effects after being exposed to a veterinary drug?

- [ ] YES
- [ ] NO

If yes, kindly fill **form Nº 3.**

Did you report them ?

- [ ] YES
- [ ] NO

If yes, to whom ?

- [ ] Hospital
- [ ] The ‘Directorate of Veterinary Service (DSV)
- [ ] Colleague
- [ ] Human pharmacy
- [ ] National veterinary Council
- [ ] Veterinary pharmacy
- [ ] Human pharmacy that sells veterinary drugs
- [ ] Local representative of the pharmaceutical industry
- [ ] Others (precise) ……….………………………………………………………………..

If no, why?

- [ ] Not sure it was due to the drug
- [ ] There are no regulations on reporting adverse effects
- [ ] Unawareness on the attitude to take
- [ ] Lack of information feedback
- [ ] Other (precise)………………………………………………………………………………

5- Have you ever heard of a case of intoxication or adverse effects occurring in a human that was exposed to a particular veterinary drug?

- □ YES
- □ NO

If yes, kindly fill **form Nº 3**

6- In your opinion, is it important to report cases of adverse effects or suspected cases of lack of efficacy of veterinary drug ?

- □ YES
- □ NO

If yes, why ?

- □ Establishment of a cause-and-effect relationship between the uses of a drug and the occurrence of adverse effects
- □ Permits that corrective action be taken by the competent authorities for the improvement of veterinary drugs on the market
- □ Protection of animal health and public health
- □ Withdrawal of defective drugs from the market
- □ Provision of care for victims
- □ Environmental protection
- □ Reassessment of the risk/benefit balance of the incriminated drugs
- □ Others (precise) ……………………………………………………………………………………

If no, why?

- □ Veterinary drug misuse
- □ Uncertainty
- □ Other(precise)………………………………………………………………………………………
- □

<u>Form Nº1 :</u> Cases of serious or unexpected adverse effects observed in animals and possibly due to a veterinary drug

Record N°…..
Date :……/…../……….
Questionnaire N° : ……

Year	Name of drug	Active ingredient(s)	Used as on the leaflet	Animal species	Number	The adverse effects observed were:	How the disease evolved	
							Evolution type	Number
			□ Yes<br>□ No<br>□I don't know				□Healing with no sequelae	
							□Healing with sequalae	
							□Death	
							□Unknown	
			□ Yes<br>□ No				□Healing with no sequelae	

			□ I don’t know				□Healing with sequalae	
							□Death	
							□Unknown	
			□ Yes<br>□ No<br>□ I don’t know				□Healing with no sequelae	
							□Healing with sequalae	
							□Death	
							□Unknown	
			□ Yes<br>□ No<br>□ I don’t know				□Healing with no sequelae	
							□Healing with sequalae	
							□Death	
							□Unknown	

<u>Form Nº2 : C</u>ases of suspected drug inefficacy with respect to the expected efficacy on an animal and possibly due to a veterinary drug

Record N°…..
Date :……/…../ ………….
Questionnaire N° : ……

Year	Name of drug	Active ingredient(s)	Used as on the leaflet	Animal species	Number	Inefficacy was suspected upon observing the following:	How the disease evolved	
							Evolution type	Number
			□ Yes<br>□ No<br>□ I don’t know			□ Insufficient protection from disease<br>□ Intensification of disease or symptoms<br>□ An insufficient therapeutic effect after administering the usual doses<br>□ No therapeutic effect<br>□ Others	□Healing with no sequelae	
							□Healing with sequalae	
							□Death	
							□Unknown	
							□Healing without sequelae after treatment was changed	
							□Healing with sequelae after treatment was changed	
			□ Yes<br>□ No<br>□ I don’t know			□ Insufficient protection from disease<br>□ Intensification of disease or symptoms	□Healing with no sequelae	
							□Healing with sequalae	

						□ An insufficient therapeutic effect after administering the usual doses<br>□ No therapeutic effect<br>□ Others	□Death	
							□Unknown	
							□Healing without sequelae after treatment was changed	
							□Healing with sequelae after treatment was changed	

Form No3 : Case of intoxication or adverse effects observed in human and possibly ascribed to a veterinary drug

Record N°…..
Date :……/…../…….
Questionnaire N° : ……

Year	Case N°	Sex	Age	Occupation	Product involved	Route of exposure	Adverse effects	Evolution of the disease	
								Evolution type	Number
					Name…………..<br>Pharmaceutical formulation……<br>Active ingredient(s) …………………<br>Pharmaceutical class ………..			□Healing with no sequelae	
								□Healing with sequalae	
								□Death	
								□Unknown	
					Name…………..<br>Pharmaceutical formulation……<br>Active ingredient(s) …………………<br>Pharmaceutical class ……………			□Healing with no sequelae	
								□Healing with sequalae	
								□Death	
								□Unknown	
					Name…………..<br>Pharmaceutical formulation……<br>Active ingredient(s) …………………<br>Pharmaceutical class ………..			□Healing with no sequelae	
								□Healing with sequalae	
								□Death	
								□Unknown	
